# Impact of persistent endotracheal tube biofilm on ventilator-associated pneumonia clinical and microbiological response

**DOI:** 10.1186/2197-425X-3-S1-A700

**Published:** 2015-10-01

**Authors:** M Gordon Sahuquillo, P Geffner, M Aroca, E Villarreal Tello, J Ruiz Ramos, B Ruiz Orenga, MA Sanchez Lopez, J Frasquet, E Gonzalez Barbera, A Castellanos Ortega, P Ramirez Galleymore

**Affiliations:** Critical Care Medicine, Hospital Universitari i Politècnic La Fe, Valencia, Spain; Microbiology, Hospital Universitari i Politècnic La Fe, Valencia, Spain

## Introduction

Ventilator-associated pneumonia (VAP) is the most common infectious complication in critically ill patients. The formation of biofilm on endotracheal tube (ETT) surface plays an important role in pathogenesis of this infection, because it facilitates the growth and survival of microorganisms and difficults the action of antimicrobial therapy.

## Objectives

The aim of this study is to evaluate the influence of the persistence of viable microorganisms in the ETT biofilm on clinical and microbiological response to VAP.

## Methods

All patients over 18 years old admitted to the Intensive Care Unit of the Hospital Universitario and Politecnico la Fe (Valencia, Spain), undergoing mechanical ventilation and with VAP diagnosis were included. Clinical and microbiological data were collected at the moment of inclusion and on days 3 and 7 after VAP diagnosis. After ETT removal, ETT was washed and a piece of 0.5 cm length was cut and sonicated and the resulting material was quantitatively cultured (100 uL blood agar plate with counting of colonies with 100 uL in isolation in chocolate blood agar). Clinical response (resolution of VAP signs: fever, high leucocyte count and respiratory purulence) was evaluated at day 3 and day 7. Median and interquartile range (IR) was calculated for continuous variables, and absolute and relative frequencies for discrete variables. Statistical analysis was done by chi-square test.

## Results

23 patients with VAP diagnosis were included, 60% men, age 55 years (IR: 51 - 67), APACHE II 22 (IR: 15 - 25). The median of days of mechanical ventilation was 17 days (IR: 13 - 20) and until VAP diagnosis was 11 days (IR: 6 - 14). The most common etiological agents were gram-negative bacilli producers of extended-spectrum beta-lactamases (ESBL) (43.6%), multidrug resistant (MDR) *P. aeruginosa* (17.4%), MDR *A. baumannii* (17.4%) and methicillin-sensitive *S. aureus* (17.4%). 83% of patients received an appropriate empiric antibiotic treatment. Median SOFA scale at VAP diagnosis was 5 (IR: 3 - 8). EET biofilm (persistence of viable microorganisms after sample sonication) was detected in 61% of the cases. No differences were observed according to the appropriateness of initial empiric treatment (78.6% vs. 80%; p 0.728) or the use of inhaled antibiotics such as tobramycin or colistin (42.8% vs. 20%; p 0.366). ETT biofilm was associated with lower percentages of clinical response on days 3 (7% vs. 20 %; p 0.468) and day 7 (21.4% vs. 60%; p 0.176).

## Conclusion

Persistence of viable and potentially infectious microorganisms on ETT biofilm is a common phenomenon after VAP and is not influenced by the use of systemic or inhaled antibiotics. Although our sample size does not allow drawing conclusions, the microbiological persistence seems to be associated with a worse VAP clinical response.
